# Growth Profile and Endocrine Dysfunction in Children Aged 2-10 Years With Transfusion-Dependent β-Thalassemia Major

**DOI:** 10.7759/cureus.116572

**Published:** 2026-09-20

**Authors:** Ashutosh Tripathi, Priyanka Singh, Archana Bundela, Suresh N Singh, Rachita Chatterjee, Vijay K Singh

**Affiliations:** 1 Pediatrics and Neonatology, Baba Raghav Das Medical College, Gorakhpur, IND; 2 Pediatrics, Baba Raghav Das Medical College, Gorakhpur, IND; 3 Pathology, Baba Raghav Das Medical College, Gorakhpur, IND; 4 Pediatric Medicine, Baba Raghav Das Medical College, Gorakhpur, IND

**Keywords:** endocrine dysfunction, growth impairment, iron chelation therapy, serum ferritin levels, transfusion-dependent thalassemia (tdt), transfusion-related iron overload, β-thalassemia major

## Abstract

Background

Advances in regular blood transfusion and iron chelation therapy have markedly improved survival in children with transfusion-dependent β-thalassemia major (TDT). As survival has increased, growth impairment and endocrine disorders related to chronic iron overload have become increasingly important causes of long-term morbidity. This study examined the prevalence of endocrine abnormalities and their relationship with growth parameters and iron overload in children with TDT.

Methods

A hospital-based cross-sectional study was conducted among 150 children aged 2-10 years with TDT attending a tertiary care center in northern India. Clinical details, anthropometric measurements, serum ferritin concentrations, and endocrine investigations, including thyroid function, glycemic profile, gonadal hormones, growth hormone, cortisol, calcium, parathyroid hormone, and vitamin D levels, were recorded. Correlation analyses and multivariable logistic regression were used to explore factors associated with endocrine dysfunction.

Results

Overall, 78.7% of children had at least one endocrine abnormality. Thyroid dysfunction (63.3%) and vitamin D deficiency (62.0%) were the most frequent disorders, followed by gonadal dysfunction (46.7%), serum cortisol abnormalities (41.3%), and low random growth hormone levels (38.7%). Older children had a greater burden of endocrine dysfunction than younger children (all p<0.001). Serum ferritin showed positive correlations with thyroid-stimulating hormone, follicle-stimulating hormone, luteinizing hormone, and glycated hemoglobin, while negative correlations were observed with height-for-age Z-score, weight-for-age Z-score, free thyroxine, and serum cortisol (all p<0.001). Increasing age (adjusted OR: 1.32), higher serum ferritin levels (adjusted OR: 1.12 per 100 ng/mL increase), longer duration of transfusion therapy, and poor adherence to chelation therapy independently predicted endocrine dysfunction.

Conclusions

Endocrine abnormalities are common even in young children with TDT and are closely associated with increasing iron burden. Early endocrine evaluation, effective transfusion practices, good adherence to iron chelation therapy, and regular monitoring of iron overload may help reduce endocrine complications and support better growth outcomes.

## Introduction

β-thalassemia major is one of the most prevalent inherited hemoglobin disorders worldwide. It is characterized by defective or absent synthesis of β-globin chains, resulting in severe anemia, ineffective erythropoiesis, and lifelong transfusion dependence [1]. Although survival has been dramatically improved by regular blood transfusions, repeated transfusions result in progressive iron overload, which remains the major cause of long-term morbidity and mortality in these patients [2,3].

Iron chelation therapy has improved survival significantly in children with transfusion-dependent β-thalassemia major (TDT). Consequently, chronic complications such as growth failure and endocrine dysfunction have emerged as important determinants of health-related quality of life [3,4]. Excess iron deposition in endocrine organs such as the pituitary, thyroid, pancreas, gonads, and parathyroid glands disrupts hormonal homeostasis and predisposes the affected children to short stature, delayed puberty, hypothyroidism, hypogonadism, impaired glucose tolerance, diabetes mellitus, and metabolic bone disease [4,5].

Growth retardation is one of the most common complications of thalassemia major with multifactorial etiology. Chronic anemia, tissue hypoxia, nutritional deficiencies, iron overload, and chelation-related toxicity all impair linear growth and delay maturation [6,7]. Endocrine abnormalities are reported to be present in 30%-70% of patients with TDT, and the prevalence increases with age, duration of transfusion therapy, and cumulative iron burden [4,8].

India bears a major share of the burden of β-thalassemia, constituting nearly one-fourth of the global disease burden [9]. However, data on growth pattern and endocrine dysfunction in younger children with TDT are limited, especially from north India. Early diagnosis of endocrine disorders is important because early treatment may prevent irreversible complications and improve long-term outcome [10,11]. Hence, our study was carried out to study the growth pattern and endocrine dysfunction in children with TDT in the age group of 2-10 years receiving regular transfusions and oral chelation therapy and their relation to iron overload and disease-related factors.

Few studies from northern India have evaluated both growth parameters and a complete endocrine profile in children with TDT between 2 and 10 years of age. This study provides region-specific data and studies the association of iron overload and multiple endocrine abnormalities in this vulnerable age group.

## Materials and methods

This cross-sectional study was conducted in the Department of Pediatrics, Baba Raghav Das (BRD) Medical College, Gorakhpur, Uttar Pradesh, India, from January 2025 to December 2025. The study aimed to determine the prevalence and pattern of endocrine dysfunction and their association with growth parameters and iron overload among children with TDT.

All children were enrolled as per eligibility criteria (Table 1).

**Table 1:** Eligibility of children

Inclusion criteria	Exclusion criteria
Children aged 2-10 years with confirmed β-thalassemia major diagnosed by hemoglobin electrophoresis or high-performance liquid chromatography	Children with other chronic systemic illnesses or congenital disorders affecting growth or endocrine function
Receiving regular blood transfusions every 2-4 weeks	Patients not receiving regular transfusion or chelation therapy
Receiving oral iron chelation therapy (deferasirox or deferiprone)	Children whose parents or guardians declined participation
Parents or legal guardians willing to provide written informed consent	-----------

Sample size calculation

The sample size was calculated using the formula for estimating a single population proportion:

 \[ n = \frac{Z^{2} \times p \times q}{d^{2}} \]

Where: n = required sample size, Z = 1.96 (95% confidence level), p = expected prevalence of endocrine dysfunction, q = 1 - p, d = absolute precision (margin of error). Expected prevalence [8] (p) = 70% (0.70), q = 0.30, Z = 1.96, and absolute precision (d) = 7.5% (0.075). Allowing for approximately 5% incomplete or missing data. The required minimum sample size was 145; therefore, 150 children were enrolled.

Data collection

A predesigned and pretested case record form was used to collect information regarding age, sex, residence, socioeconomic status (modified Kuppuswamy scale [12]), age at diagnosis and duration of disease, frequency of blood transfusions, type, duration, and compliance with iron chelation therapy, and family history and treatment records. Clinical records were reviewed to obtain pre-transfusion hemoglobin values and serum ferritin levels from the previous six visits, and their mean values were calculated.

Anthropometric assessment

Anthropometric measurements were performed by trained personnel using standardized techniques. Weight was measured to the nearest 0.1 kg using a calibrated digital weighing scale, and height to the nearest 0.1 cm using a stadiometer, with the child standing barefoot in the Frankfurt plane. Body mass index (BMI) was calculated as weight (kg)/height (m²).

Height-for-age Z-score (HAZ), weight-for-age Z-score (WAZ), and BMI-for-age Z-score (BAZ) were calculated using the WHO Child Growth Standards (WHO Anthro) for children aged <5 years and the WHO Growth Reference 5-19 years (WHO AnthroPlus) for children aged ≥5 years [13].

Endocrine test results were interpreted using age- and sex-specific pediatric reference intervals as recommended in Nelson Textbook of Pediatrics [14].

All children underwent clinical examination and laboratory evaluation for endocrine abnormalities. The following investigations were performed (Table 2).

**Table 2:** Assessment of endocrine dysfunction *As growth hormone stimulation testing was not performed, these findings were considered suggestive of low random serum growth hormone. FSH, follicle-stimulating hormone; LH, luteinizing hormone; TSH, thyroid-stimulating hormone.

Endocrine parameters	Endocrine dysfunction definition
Thyroid function tests	Abnormal TSH and/or T4/T3 levels
Glycated hemoglobin (HbA1c)	Elevated HbA1c according to American Diabetes Association criteria
Gonadal hormones	Abnormal FSH, LH, or testosterone levels
Growth hormone and serum cortisol levels	*Low random serum growth hormone level was defined as a serum growth hormone concentration below the laboratory reference range, abnormal serum cortisol level.
Vitamin D deficiency	Serum 25-hydroxyvitamin D level <20 ng/mL

Quality control

All anthropometric measurements were obtained by trained investigators using calibrated equipment. Laboratory investigations were performed in the central biochemistry laboratory using a standardized method on an ARCHITECT i1000SR immunoassay analyzer (Abbott Laboratories, Abbott Park, Illinois, USA), employing chemiluminescent microparticle immunoassay technology according to the manufacturer's instructions and internal quality control procedures. Data were entered independently by two investigators and cross-verified to minimize transcription errors. All case record forms were checked for completeness before analysis.

Statistical analysis

We analyzed the data by using the Statistical Package for Social Sciences (SPSS) version 26.0 (IBM Corp., Armonk, NY, USA). Continuous variables were expressed as mean ± standard deviation, while categorical variables were presented as frequencies and percentages. Comparisons between groups were performed using Student's t-test or Mann-Whitney U test for continuous variables and chi-square test or Fisher's exact test for categorical variables. Pearson's or Spearman's correlation coefficients were used to assess the relationship between serum ferritin and growth or endocrine parameters. Multivariable logistic regression analysis was performed to identify independent predictors of endocrine dysfunction. A p-value <0.05 was considered statistically significant.

Ethical considerations

The study was approved by the Institutional Ethics Committee of Baba Raghav Das Medical College, Gorakhpur, Uttar Pradesh, India, vide letter No. 295(K)/IHEC/2025. Written informed consent was obtained from parents or legal guardians before enrollment, and the confidentiality of all children was maintained throughout the study.

## Results

A total of 150 children with TDT were enrolled. Most participants were aged 5-10 years (69.3%), with a slight male predominance (56.0%). The majority belonged to the upper lower (34.7%) or lower middle (29.3%) socioeconomic classes. The mean serum ferritin concentration was 1678 ± 312 ng/mL, and the mean vitamin D level was 21.6 ± 8.2 ng/mL. Detailed baseline demographic and clinical characteristics are presented in Table 3.

**Table 3:** Baseline characteristics of study children Values are presented as frequency (%) or mean ± standard deviation (SD).

Variable	Value
Age group, 2-5 years, n (%)	46 (30.7)
Age group, 5-10 years, n (%)	104 (69.3)
Male sex, n (%)	84 (56.0)
Female sex, n (%)	66 (44.0)
Upper lower socioeconomic class, n (%)	52 (34.7)
Lower middle socioeconomic class, n (%)	44 (29.3)
Age at diagnosis of 6-12 months, n (%)	72 (48.0)
Duration of chelation therapy of 5-7 years, n (%)	58 (38.7)
Good compliance with chelation therapy, n (%)	112 (74.7)
Serum ferritin (ng/mL), mean ± SD	1678 ± 312
Vitamin D level (ng/mL), mean ± SD	21.6 ± 8.2

Growth assessment revealed considerable impairment among the study children. The mean height, weight, and BMI were 104.8 ± 13.6 cm, 17.8 ± 5.4 kg, and 16.0 ± 2.1 kg/m², respectively. The mean HAZ, WAZ, and BAZ were −1.84 ± 1.12, −1.62 ± 1.08, and −0.82 ± 0.94, respectively. Overall, 42.0% of children were stunted, 36.7% were underweight, and 19.3% had wasting, indicating a substantial burden of growth impairment in children with TDT despite regular transfusion and chelation therapy (Table 4).

**Table 4:** Growth parameters of children with transfusion-dependent β-thalassemia major Growth indices were calculated using WHO Child Growth Standards (WHO Anthro) for children aged <5 years and WHO Growth Reference (WHO AnthroPlus) for children aged ≥5 years.

Growth parameter	Value
Height (cm), mean ± SD	104.8 ± 13.6
Weight (kg), mean ± SD	17.8 ± 5.4
Body mass index (kg/m²), mean ± SD	16.0 ± 2.1
Height-for-age Z-score (HAZ), mean ± SD	−1.84 ± 1.12
Weight-for-age Z-score (WAZ), mean ± SD	−1.62 ± 1.08
BMI-for-age Z-score (BAZ), mean ± SD	−0.82 ± 0.94
Stunting (HAZ < −2 SD), n (%)	63 (42.0)
Severe stunting (HAZ < −3 SD), n (%)	22 (14.7)
Underweight (WAZ < −2 SD), n (%)	55 (36.7)
Severe underweight (WAZ < −3 SD), n (%)	18 (12.0)
Wasting (BAZ < −2 SD), n (%)	29 (19.3)
Severe wasting (BAZ < −3 SD), n (%)	8 (5.3)

All children required regular blood transfusions and were receiving oral deferasirox therapy. Nearly half of the children were diagnosed between 6 and 12 months of age (48.0%), while 34.7% and 17.3% were diagnosed between 13-24 months and 25-36 months, respectively. The most common duration of chelation therapy was 5-7 years (38.7%). Good compliance with chelation therapy was observed in 74.7% of children, whereas 25.3% had poor compliance (Table 5).

**Table 5:** Clinical and treatment characteristics of children with transfusion-dependent β-thalassemia major

Variable	Category	n (%)
Age at diagnosis (months)	6-12	72 (48.0)
	13-24	52 (34.7)
	25-36	26 (17.3)
Duration of chelation therapy (years)	2-4	42 (28.0)
	5-7	58 (38.7)
	8-10	50 (33.3)
Regular transfusion requirement	Yes	150 (100.0)
Chelation compliance	Good	112 (74.7)
	Poor	38 (25.3)
Chelation therapy received	Deferasirox	150 (100.0)

Endocrine abnormalities were identified in 78.7% of children. Thyroid dysfunction (63.3%) and vitamin D deficiency (62.0%) were the most common abnormalities, followed by gonadal dysfunction (46.7%), serum cortisol abnormalities (41.3%), low random growth hormone levels (38.7%), and elevated glycated hemoglobin (HbA1c) levels (13.3%) (Figure 1).

**Figure 1:**
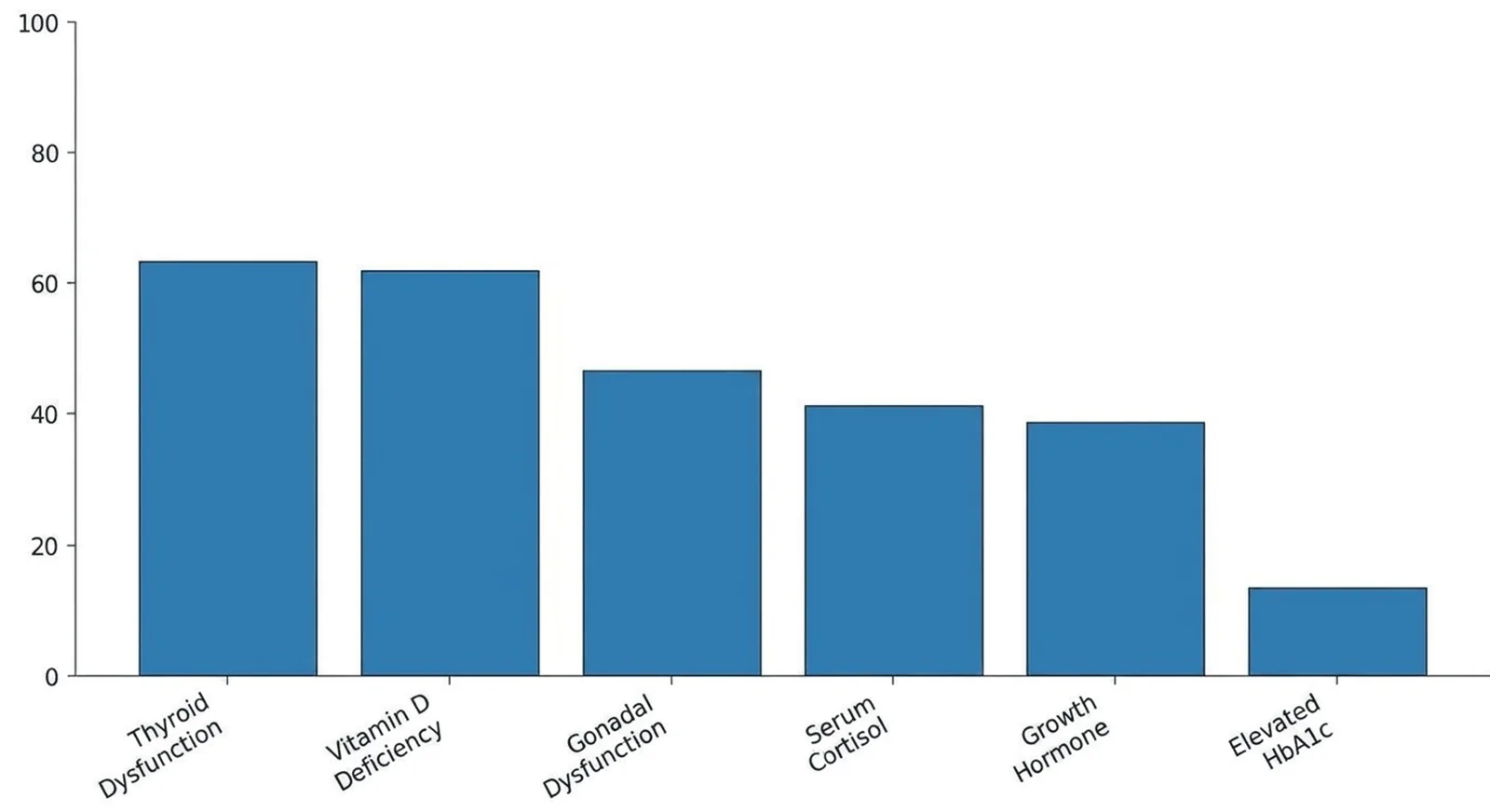
Prevalence of endocrine abnormalities in children with transfusion-dependent β-thalassemia major HbA1c, glycated hemoglobin.

Compared with children aged 2-5 years, those aged 5-10 years had significantly higher thyroid-stimulating hormone (TSH), follicle-stimulating hormone (FSH), and luteinizing hormone (LH) levels and lower triiodothyronine (T3), tetraiodothyronine (T4), growth hormone, HbA1c, and serum cortisol levels (all p<0.001) (Table 6).

**Table 6:** Comparison of endocrine parameters according to age group *p-value < 0.05 is considered statistically significant. Endocrine test results were interpreted using age- and sex-specific pediatric reference intervals as recommended in Nelson Textbook of Pediatrics. FSH, follicle-stimulating hormone; HbA1c, glycated hemoglobin; LH, luteinizing hormone; T3, triiodothyronine; T4, tetraiodothyronine; TSH, thyroid-stimulating hormone.

Parameter	2-5 years (n=46), mean ± SD	5-10 years (n=104), mean ± SD	p-value
TSH (mIU/L)	3.18 ± 1.02	6.42 ± 2.36	<0.001*
T3 (ng/dL)	118 ± 26	82 ± 22	<0.001*
T4 (ng/dL)	1.22 ± 0.24	0.86 ± 0.26	<0.001*
HbA1c (%)	5.8 ± 1.0	6.2 ± 1.1	<0.001*
FSH (mIU/mL)	1.92 ± 0.82	4.24 ± 1.86	<0.001*
LH (mIU/mL)	1.34 ± 0.62	3.48 ± 1.54	<0.001*
Low random growth hormone level (ng/mL)	7.82 ± 2.16	4.12 ± 1.86	<0.001*
Serum cortisol (µg/dL)	14.6 ± 3.2	9.2 ± 2.8	<0.001*

Heat map illustrating the strength and direction of Pearson's correlation coefficients (r) between serum ferritin levels and clinical, growth, and endocrine parameters in children with TDT. Warmer colors represent positive correlations, whereas cooler colors indicate negative correlations. Serum ferritin demonstrated significant positive correlations with age (r=0.442), duration of chelation therapy (r=0.486), TSH (r=0.496), HbA1c (r=0.468), FSH (r=0.384), and LH (r=0.402). In contrast, significant negative correlations were observed with HAZ (r = −0.350), WAZ (r = −0.300), free T4 (r = −0.452), and serum cortisol (r = −0.436) (all p<0.001). No significant correlation was observed between serum ferritin and BAZ. These findings suggest that increasing iron overload is associated with impaired growth and progressive endocrine dysfunction (Figure 2).

**Figure 2:**
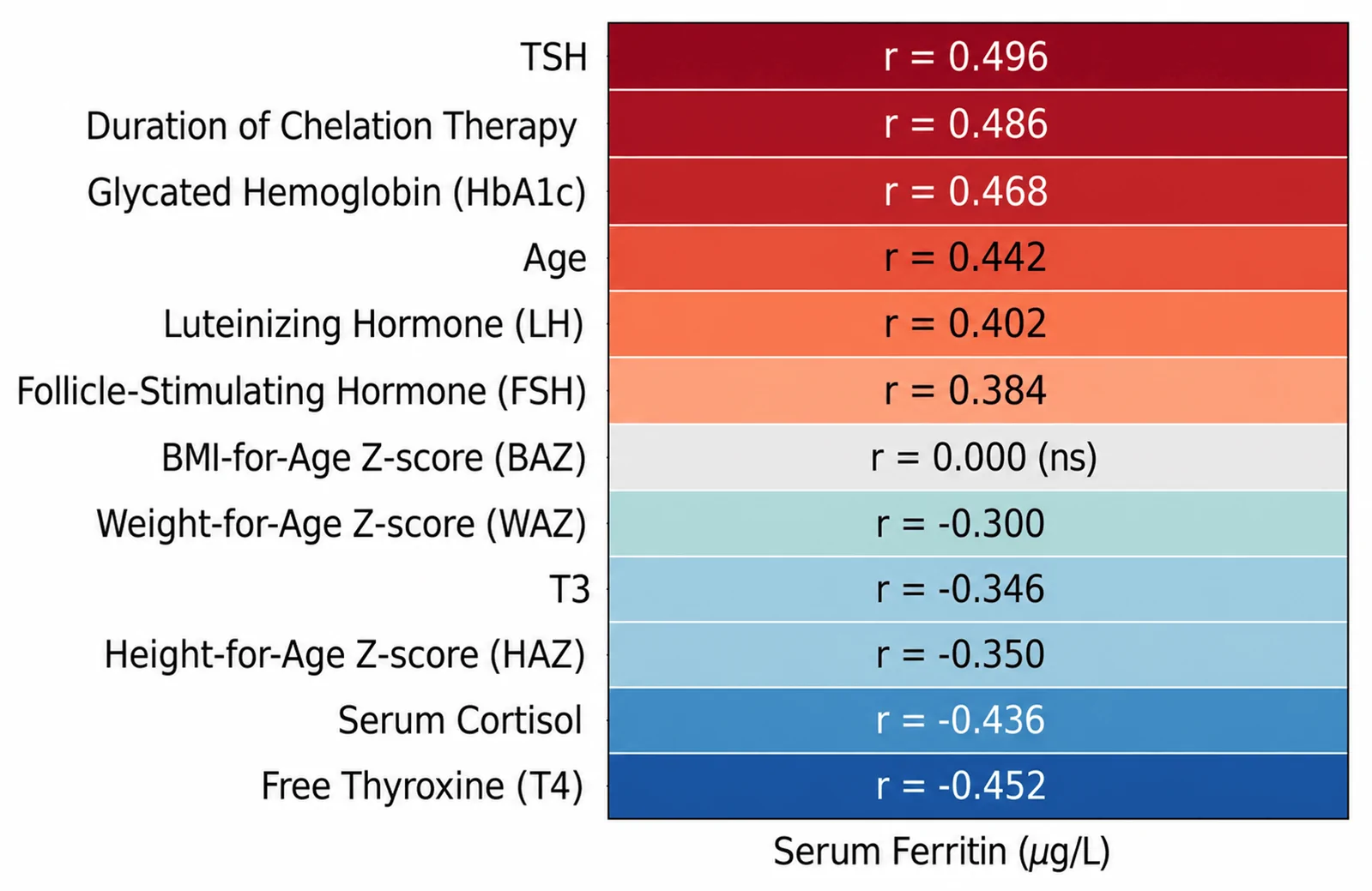
Serum ferritin correlations Color intensity indicates the Pearson correlation coefficient (r), ranging from deep blue (negative correlation) to deep red (positive correlation). Serum ferritin demonstrates significant correlations across all assessed clinical, anthropometric (HAZ, WAZ, BAZ), and endocrine parameters. ns, non-significant; T3, triiodothyronine; TSH, thyroid-stimulating hormone.

Multivariable logistic regression analysis identified increasing age (adjusted OR: 1.32, 95% CI: 1.08-1.61; p=0.006), higher serum ferritin levels (adjusted OR: 1.12 per 100 ng/mL increase, 95% CI: 1.05-1.20; p<0.001), longer duration of transfusion therapy (adjusted OR: 1.21, 95% CI: 1.04-1.40; p=0.011), and poor chelation compliance (adjusted OR: 2.48, 95% CI: 1.18-5.23; p=0.016) as independent predictors of endocrine dysfunction. Higher pre-transfusion hemoglobin was independently associated with lower odds of endocrine dysfunction (adjusted OR: 0.76, 95% CI: 0.61-0.95; p=0.018), whereas sex was not significantly associated with endocrine dysfunction (adjusted OR: 1.18, 95% CI: 0.64-2.18; p=0.591) (Figure 3).

**Figure 3:**
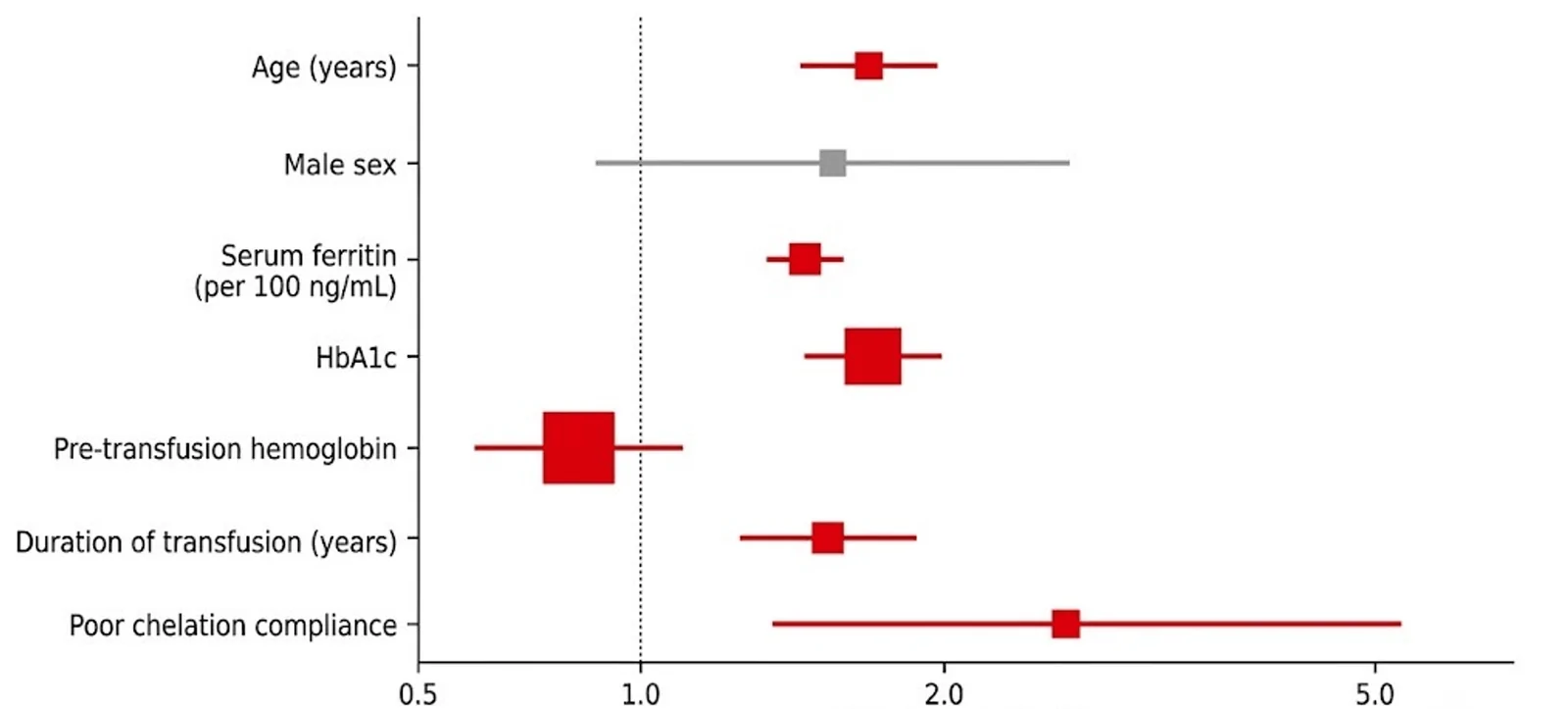
Forest plot of independent predictors of endocrine dysfunction in children with transfusion-dependent β-thalassemia major HbA1c, glycated hemoglobin.

Children with endocrine abnormalities had significantly higher mean serum ferritin levels than those without the corresponding abnormalities. The strongest associations were observed for gonadal dysfunction and low random growth hormone levels, supporting a relationship between increasing iron overload and endocrine complications (Table 7).

**Table 7:** Comparison of serum ferritin levels according to specific endocrine abnormalities *p-value < 0.05 is considered statistically significant.

Endocrine abnormality	Ferritin present (mean ± SD)	Ferritin absent (mean ± SD)	p-value
Thyroid dysfunction	1850 ± 420	1410 ± 310	<0.001*
Vitamin D deficiency	1760 ± 385	1515 ± 295	0.002*
Low random serum growth hormone level	1885 ± 450	1540 ± 330	<0.001*
Gonadal dysfunction	1910 ± 435	1495 ± 310	<0.001*

## Discussion

In this cohort, endocrine abnormalities were identified in nearly four-fifths of children with TDT, with thyroid dysfunction and vitamin D deficiency being the most common disorders. Increasing serum ferritin concentrations were significantly associated with endocrine dysfunction, highlighting the persistent burden of iron overload despite regular transfusions and chelation therapy. Recent international guidelines and contemporary reviews emphasize that improved survival in TDT has shifted clinical priorities toward early identification and prevention of chronic complications through regular endocrine surveillance, optimization of transfusion regimens, and individualized iron chelation therapy [3,11].

Thyroid dysfunction was the most frequent endocrine abnormality in our study, affecting approximately two-thirds of the children. Higher serum ferritin levels were significantly associated with abnormal thyroid function, supporting the role of chronic iron deposition within the thyroid gland and hypothalamic-pituitary axis. Similar findings have been reported by De Sanctis et al. and Soliman et al., who identified hypothyroidism as one of the earliest endocrine complications in chronically transfused patients [4,5]. The latest Thalassaemia International Federation (TIF) guidelines and recent endocrine screening recommendations advocate annual thyroid function assessment from early childhood because biochemical abnormalities often precede clinical manifestations, allowing timely intervention [11,15,16].

Vitamin D deficiency was observed in 62% of children, highlighting the continued burden of metabolic bone disease in TDT. Chronic iron overload, impaired hepatic vitamin D metabolism, nutritional deficiencies, and reduced physical activity are recognized contributors to poor bone health in these patients. Routine assessment of vitamin D, calcium, phosphorus, and parathyroid hormone is therefore recommended as part of comprehensive endocrine evaluation to facilitate early intervention and reduce skeletal complications [10,11,16].

Older children demonstrated significantly greater endocrine dysfunction than younger children, with higher TSH, FSH, and LH levels and lower T3, T4, serum cortisol, and random growth hormone levels. This finding is consistent with previous studies demonstrating that endocrine complications increase with age, cumulative transfusion exposure, and iron burden [3,4,8]. Contemporary evidence also supports initiating endocrine screening before the appearance of overt clinical manifestations to improve long-term outcomes [11,16].

Serum ferritin showed significant positive correlations with TSH, FSH, and LH levels and negative correlations with HAZ, WAZ, T4, and serum cortisol, indicating that increasing iron overload adversely affects both growth and endocrine function. Furthermore, multivariable logistic regression identified increasing age, higher serum ferritin levels, longer duration of transfusion therapy, and poor chelation compliance as independent predictors of endocrine dysfunction, whereas higher pre-transfusion hemoglobin was protective. These findings reinforce the importance of maintaining adequate transfusion regimens, improving adherence to iron chelation therapy, and routinely monitoring iron burden to reduce endocrine complications [11,15,17,18].

The strengths of this study include its relatively large sample size, comprehensive endocrine evaluation, standardized anthropometric assessment using World Health Organization growth standards, and evaluation of the relationship between serum ferritin and endocrine abnormalities in young children, a population for which data from northern India remain limited. However, the cross-sectional design precludes causal inference, the single-center setting may limit generalizability, and serum ferritin was used as a surrogate marker of iron overload because magnetic resonance imaging (MRI) T2* assessment was unavailable. Additionally, growth hormone stimulation testing was not performed; therefore, low random serum growth hormone levels should not be interpreted as confirmed growth hormone deficiency. Future multicenter prospective studies incorporating MRI T2* assessment, dynamic endocrine testing, and standardized endocrine screening protocols are needed to better define the natural history of endocrine complications and evaluate the long-term impact of optimized chelation therapy [16-18].

Limitations

Our study has several limitations. First, its cross-sectional design precludes the establishment of causal relationships between iron overload and endocrine dysfunction. Second, the study was conducted at a single tertiary-care center, which may limit the generalizability of the findings to the wider population of children with β-thalassemia major. Third, the relatively small sample size and inclusion of only children aged 2-10 years may have underestimated the prevalence of certain endocrinopathies, particularly delayed puberty and diabetes mellitus, which are more common in adolescents. Fourth, serum ferritin was used as a surrogate marker of iron overload; however, more accurate measures such as MRI T2* assessment of hepatic and cardiac iron were not available. Finally, the absence of a healthy control group and the lack of longitudinal follow-up limited the assessment of temporal changes in growth and endocrine function. Low random serum growth hormone levels were observed in a substantial proportion of children. However, because growth hormone stimulation testing was not performed, these findings should be interpreted cautiously and cannot be considered diagnostic of growth hormone deficiency.

## Conclusions

Children with TDT exhibit a high prevalence of endocrine dysfunction and growth impairment despite receiving regular transfusions and oral chelation therapy. Thyroid dysfunction and vitamin D deficiency were the most common endocrine abnormalities, and endocrine disturbances worsened with increasing age and iron burden. Serum ferritin showed significant associations with both growth parameters and endocrine abnormalities, highlighting the central role of chronic iron overload in the development of these complications. These findings emphasize the importance of early diagnosis, adequate transfusion practices, strict adherence to chelation therapy, and regular endocrine surveillance to facilitate early intervention, reduce endocrine morbidity, and improve long-term health outcomes and quality of life.

## References

[1] Weatherall DJ, Clegg JB (2001). The Thalassaemia Syndromes. Oxford: Blackwell Science.

[2] Modell B, Darlison M (2008). Global epidemiology of haemoglobin disorders and derived service indicators. Bull World Health Organ.

[3] Motta I, Mancarella M, Marcon A, Vicenzi M, Cappellini MD (2020). Management of age-associated medical complications in patients with β-thalassemia. Expert Rev Hematol.

[4] De Sanctis V, Soliman AT, Canatan D (2019). An ICET-A survey on occult and emerging endocrine complications in patients with β-thalassemia major: conclusions and recommendations. Acta Biomed.

[5] De Sanctis V, Soliman A, Yassin M (2013). Iron overload and glucose metabolism in subjects with β-thalassaemia major: an overview. Curr Diabetes Rev.

[6] Rathaur VK, Imran A, Pathania M (2020). Growth pattern in thalassemic children and their correlation with serum ferritin. J Family Med Prim Care.

[7] Singh P, Seth A (2017). Growth and endocrine issues in children with thalassemia. Pediatr Hematol Oncol J.

[8] Vogiatzi MG, Macklin EA, Trachtenberg FL (2009). Differences in the prevalence of growth, endocrine and vitamin D abnormalities among the various thalassaemia syndromes in North America. Br J Haematol.

[9] Colah R, Gorakshakar A, Nadkarni A (2010). Global burden, distribution and prevention of β-thalassemias and hemoglobin E disorders. Expert Rev Hematol.

[10] Soesanti F, Putriasih SA, Pulungan A, Wahidiyat PA (2013). Endocrinopathies in thalassemia major patients in thalassemia center Jakarta, Indonesia. Int J Pediatr Endocrinol.

[11] (2025). Guidelines for the Management of Transfusion-Dependent β-Thalassaemia. Nicosia, Cyprus: Thalassaemia International Federation.

[12] Mandal I, Hossain SR (2025). Modified Kuppuswamy scale updated for the year 2025. Int J Community Med Public Health.

[13] (2026). World Health Organization: Growth reference data for 5-19 years. https://www.who.int/tools/growth-reference-data-for-5to19-years/application-tools.

[14] Kliegman RM, St. Geme JW III, Blum NJ, Tasker RC, Wilson KM, Shah SS (2024). Nelson Textbook of Pediatrics.

[15] Farmaki K, Tzoumari I, Pappa C, Chouliaras G, Berdoukas V (2010). Normalisation of total body iron load with very intensive combined chelation reverses cardiac and endocrine complications of thalassaemia major. Br J Haematol.

[16] Abbas S, Khan MO, Arif A (2025). Framework for the comprehensive screening for endocrine disorders in patients with transfusion-dependent thalassemia in low-resource settings. Ann Hematol.

[17] Musallam KM, Sheth S, Cappellini MD (2026). Management of transfusion-dependent β-thalassaemia in the era of novel therapies: a prioritisation-based matrix for settings with limited resources. Lancet Haematol.

[18] Taher AT, Musallam KM, Cappellini MD, Porter JB, Farmakis D (2025). Guidelines for the Management of Transfusion-Dependent Β-Thalassaemia (TDT). Lancet Haematol.

